# Crystal structure of a tripeptide biphenyl hybrid C_50_H_56_N_6_O_10_·0.5H_2_O

**DOI:** 10.1107/S2056989020000584

**Published:** 2020-01-21

**Authors:** Thuy Quynh Le, Xuan Tu Nguyen, Hung Huy Nguyen, Dinh Hung Mac, Thai Thanh Thu Bui

**Affiliations:** a Department of Chemistry, VNU University of science, Vietnam National University, Hanoi, 19 Le Thanh Tong, Hanoi, Vietnam

**Keywords:** crystal structure, hydrogen bonding, peptide biphenyl hybrids, tripeptide Pro–Phe–Ala.

## Abstract

A peptide biphenyl hybrid compound {systematic name: dimethyl 2,2′-[((2*S*,2′*S*)-2,2′-{[(2*S*,2′*S*)-1,1′-([1,1′-biphen­yl]-2,2′-dicarbon­yl)bis­(pyrrolidine-1,2-diyl-2-carbon­yl)]bis­(aza­nedi­yl)}bis­(3-phenyl­propano­yl))bis­(aza­nedi­yl)](2*S*,2′*S*)-dipropionate hemihydrate}, C_50_H_56_N_6_O_10_·0.5H_2_O, was prepared by coupling of [1,1′-biphen­yl]-2,2′-dicarbonyl dichloride, tri­ethyl­amine and the tripeptide Pro–Phe–Ala in CH_2_Cl_2_ at 273 K under an N_2_ atmosphere. In the crystal, the asymmetric unit contains the peptide biphenyl hybrid accompanied by one-half of a water mol­ecule. A C atom of one of the proline rings is disordered between two positions in a 0.746 (11):0.254 (11) ratio. An important structural aspect of peptide compounds is their capacity to self-associate mediated by inter­molecular and intra­molecular hydrogen bonding. This characteristic can be useful in understanding the inter­actions between peptides and biomacromolecular targets, as well as to explain peptide properties.

## Abstract 2

The synthesis of the peptide biphenyl hybrid compound dimethyl 2,2′-[((2*S*,2′*S*)-2,2′-{[(2*S*,2′*S*)-1,1′-([1,1′-biphen­yl]-2,2′-dicarbon­yl)bis­(pyrrolidine-1,2-diyl-2-carbon­yl)]bis­(aza­nedi­yl)}bis­(3-phenyl­propano­yl))bis­(aza­nedi­yl)](2*S*,2′*S*)-dipropionate) is described. The crystal structure of this compound has a highly ordered supra­molecular structure with extensive inter­molecular hydrogen bonding.

## Chemical context   

Peptides are combined linear chains of amino acids and are essential for all biological processes. Consequently, they are of great interest in the biomedical field, and research into the use of peptides and modified peptides as therapeutics is increasing rapidly. At present there are over 100 approved peptide-based therapeutics on the market, with the majority being smaller than 20 amino acids (Bruno *et al.*, 2013 ▸ *)*. However, these peptides have some drawbacks: their poor absorption after oral ingestion, low diffusion in tissue organs, and low metabolic stability towards protease enzymes as well as undesired side-effects of flexible peptides due to inter­action with several receptors.

**Figure 1:**
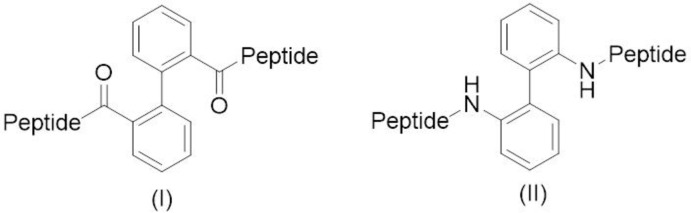
Structure of peptide-biphenyl hybrids I and II.

To overcome these disadvantages, researchers are aiming at the development of new treatment methods based on peptides and proteins, by introducing both structural and functional specific modifications and maintaining the features responsible for biological activity. The synthesis, structure, and properties of peptide–biphenyl hybrids I and II (Fig. 1 ▸), which are derivatives of 1,1-biphenyl with amino acids or peptide chains at the positions C2 and C2′ (Mann *et al.*, 2002 ▸; Montero, Mann *et al.*, 2004 ▸) have been studied intensively to overcome the disadvantages mentioned above.

The combination of biphenyl and peptide fragments provides compounds with structural (Mann *et al.*, 2002 ▸) and biological properties of significant inter­est, as illustrated by the glycopeptide anti­biotic vancomycin, the proteasome inhibitor TMC-95A (Kaiser *et al.*, 2004 ▸) and the peptide anti­biotic WS- 43708A (Rajamoorthi & Williams, 1987 ▸), aryl­omycins (Schimana *et al.*, 2002 ▸) and biphenomycins (Ezaki *et al.*, 1985 ▸). The inhibition of calpain I by biphenyl derivatives and peptide–biphenyl hybrids was reported by Montero, Albericio *et al.* (2004 ▸).

**Figure 2:**
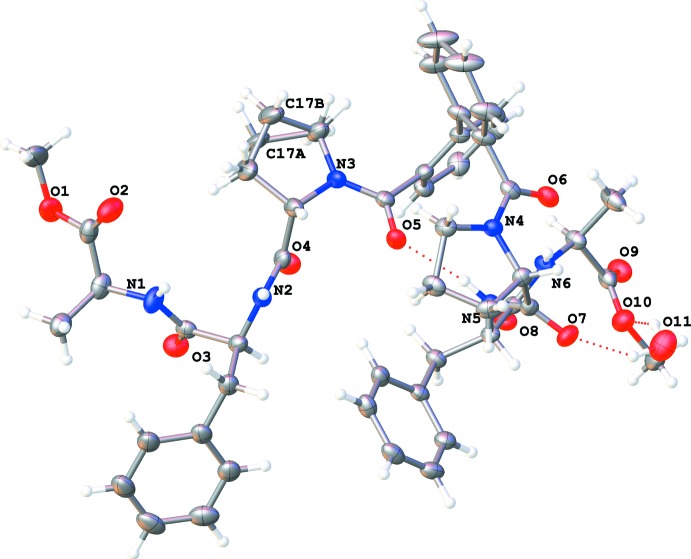
A view of the mol­ecular structure of the title compound with displacement ellipsoids drawn at the 50% probability level. H atoms are shown as small circles of arbitrary radii.

Biphenyl is a typical drug-like scaffold, which is present in 2.1% of reference drug mol­ecules (Bemis *et al.*, 1996 ▸). Based on the important role of the biphenyl unit and peptides in biological activity, we report here the synthesis and crystallographic study of a peptide–2,2′-biphenyl hybrid with the tripeptide Pro–Phe–Ala (Fig. 2 ▸).
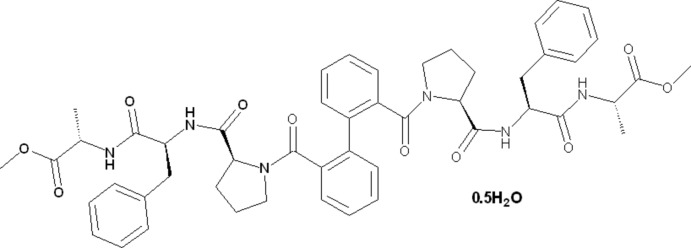


## Structural commentary   

The title compound crystallizes in space group *P*2_1_2_1_2_1_ with one mol­ecule of dimethyl 2,2′-[((2*S*,2′*S*)-2,2′-{[(2*S*,2′*S*)-1,1′-([1,1′-biphen­yl]-2,2′-dicarbon­yl)bis­(pyrrolidine-1,2-diyl-2-carbon­yl)]bis­(aza­nedi­yl)}bis­(3-phenyl­propano­yl))bis­(aza­ne­di­­yl)](2*S*,2′*S*)-dipropionate) and one-half of a water mol­ecule in the asymmetric unit (Fig. 2 ▸). One of the proline rings is disordered over two conformations and atom C17 was refined using a split model with occupancies of 0.746 (11) and 0.254 (11). An intra­molecular hydrogen bond is formed between the NH and CO groups of the two tripeptides with a distance of 2.04 Å (N5—H5⋯O5=C19, see Table 1 ▸, Fig. 2 ▸), which is slightly shorter than previously reported (Ranganathan *et al.*, 1997 ▸). The C20–C25 and C26–C31 benzene rings are roughly perpendicular to each other, with a dihedral angle between them of 84.4 (4)°. An inter­esting feature is the non-coplanarity between each phenyl ring and the C=O function of the attached peptide bond. The C26—C31—C32=O6, C26—C31—C32—N4 and C25—C20—C19=O5, C25—C20—C19—N3 torsion angles are 59.8 (4)°, −123.0 (3)° and −85.9 (4)°, −96.8 (4)°, respectively. The torsion angles ω, Φ and Ψ along the two tripeptide backbones are given in Table 2 ▸. The torsion angles φ and ψ of amino acids Phe2, Pro4, Phe5 and Ala6 (as defined in Table 2 ▸) correspond with the α region in a Ramachandran plot, while for amino acids Pro1 and Ala3 the β region is observed.

## Supra­molecular features   

**Figure 3:**
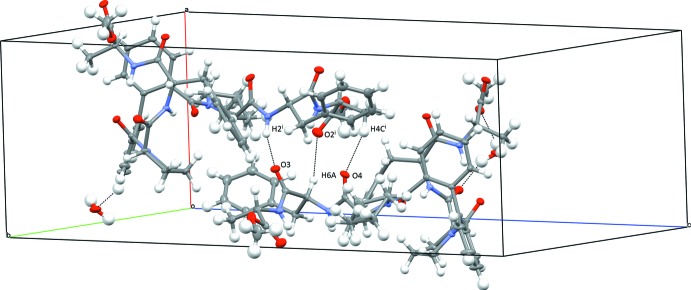
Partial crystal packing of the title compound with dashed lines representing the hydrogen bonds (see also Table 1 ▸).

The crystal packing is dominated by hydrogen bonding (Table 1 ▸). The water mol­ecule stabilizes the packing by bridging atoms O10 and O7 (hydrogen bonds O11—H11*B*⋯O10, O11—H11*A*⋯O7) and makes an additional hydrogen bond C29—H29⋯O11 with a neighbouring mol­ecule. The mol­ecules are further linked *via* a hydrogen bond between the NH and CO groups of peptide bonds (N2—H2⋯O3=C5), resulting in chains running in the *a*-axis direction (Fig. 3 ▸). In addition, five C–H⋯O=C inter­actions with H⋯O distances ranging from 2.41 to 2.67 Å are observed.

## Database survey   

A search in the Cambridge Structural Database (CSD, Version 5.40, 2019.2; Groom *et al.*, 2016 ▸) for a peptide–biphenyl hybrid with three amino acids gave no hits. We found nine structures of peptide–biphenyl hybrids containing one and two amino acids. In three of them a di­sulfide bridge is present. Three structures contain only one amino acid (MULLOU, Mann *et al.*, 2002 ▸; WAFRUR and WAFSAY, Herradón *et al.*, 2004 ▸) and two structures contain two amino acids (MULLUA, Mann *et al.*, 2002 ▸; WAFSEC, Herradón *et al.*, 2004 ▸). For the structures of MULLUA and WAFSEC, the torsion angles ϕ and ψ are located in different regions of the Ramachandran plot compared to the title structure.

## Synthesis and crystallization   

To a round-bottom flask was added amine HN–proline–phenyl­alanine–alanine–COOMe (1 eq.), Et_3_N (2 eq.) and anhydrous CH_2_Cl_2_ (50mL). To this solution was added a solution of (1,1′-biphen­yl)-2,2′-dicarbonyl dichloride in CH_2_Cl_2_ at 273 K under an N_2_ atmosphere. After completion of the reaction, the mixture was washed with 1*N* HCl solution, water and a solution of brine, respectively. The organic phase was dried over Na_2_SO_4_, filtered and evaporated under reduced pressure. The crude product was then purified by flash chromatography (AcOEt/hexane 3:2) to give a yellow solid (63% yield). The compound was recrystallized by slow evaporation in methanol to give crystals suitable for X-ray diffraction.

^1^H NMR (500 MHz, CDCl_3_, δ in ppm) δ 7.96 (*s*, 1H), 7.63 (*d*, *J* = 21.1 Hz, 1H), 7.56–7.28 (*m*, 7H), 7.32–7.07 (*m*, 11H), 6.90 (*s*, 1H), 6.84 (*d*, *J* = 7.1 Hz, 1H), 5.91 (*s*, 1H), 4.59–4.36 (*m*, 3H), 4.36–4.14 (*m*, 3H), 3.75–3.62 (*m*, 6H), 3.60–3.54 (*m*, 2H), 3.48–3.10 (*m*, 4H), 2.41 (*s*, 1H), 2.18 (*s*, 1H), 2.02–1.89 (*m*, 2H), 1.89–1.64 (*m*, 8H), 1.56 (*s*, 1H), 1.43 (*s*, 1H), 1.34–1.14 (*m*, 6H).

^13^C NMR (126 MHz, CDCl_3_, δ in ppm) δ 73.22, 172.49, 172.33, 171.45, 170.92, 170.60, 170.02, 169.43, 138.29, 137.02, 131.39, 131.03, 129.81, 129.71, 129.61, 129.41, 129.32, 128.97, 128.65, 128.50, 127.96, 127.66, 126.93, 126.63, 60.08, 58.57, 55.32, 52.37, 50.38, 48.13, 47.44, 39.20, 36.31, 32.00, 29.83, 28.58, 25.63, 24.63, 23.23, 18.56, 18.47, 18.14.

## Refinement   

Crystal data, data collection and structure refinement details are summarized in Table 3 ▸. All H atoms were positioned geometrically and treated as riding on their parent atoms with N—H = 0.88 Å and *U*_iso_(H) = 1.2*U*_eq_ (N), C_aromatic_—H = 0.95 Å and *U*_iso_(H) = 1.2*U*_eq_(C), C_proline, methylen_—H = 0.99 Å and *U*_iso_(H) = 1.2 *U*_eq_(C), C_meth­yl_—H = 0.98 Å and *U*_iso_(H) = 1.5 *U*_eq_(C), O_water_—H = 0.87 Å and *U*_iso_(H) = 1.52*U*_eq_(O). A rotating group model (AFIX 137) was applied to the methyl groups at C1, C4, C48, C50. The solvent water mol­ecule is disordered and was refined with a site occupation factor fixed to 0.5. The ring of one of the proline residues shows two conformations with refined occupancy factors for atom C17 converging to 0.746 (11) and 0.254 (11).

## Supplementary Material

Crystal structure: contains datablock(s) I. DOI: 10.1107/S2056989020000584/vm2226sup1.cif

Structure factors: contains datablock(s) I. DOI: 10.1107/S2056989020000584/vm2226Isup2.hkl

Supporting information file. DOI: 10.1107/S2056989020000584/vm2226Isup3.cdx

Supporting information file. DOI: 10.1107/S2056989020000584/vm2226Isup7.cdx

CCDC reference: 1978230

Additional supporting information:  crystallographic information; 3D view; checkCIF report

## Figures and Tables

**Table 1:** Hydrogen-bond geometry (Å, °)

*D*—H⋯*A*	*D*—H	H⋯*A*	*D*⋯*A*	*D*—H⋯*A*
O11—H11*B*⋯O10	0.87	2.15	2.899 (6)	145
O11—H11*A*⋯O7	0.87	2.03	2.804 (6)	145
C29—H29⋯O11^i^	0.95	2.57	3.339 (7)	138
N5—H5⋯O5	0.88	2.04	2.892 (3)	163
N2—H2⋯O3^ii^	0.88	2.11	2.867 (4)	143
C6—H6*A*⋯O2^iii^	1.00	2.67	3.647 (4)	165
C4—H4*C*⋯O4^ii^	0.98	2.55	3.480 (4)	159
C27—H27⋯O9^iv^	0.95	2.41	3.327 (5)	162

Symmetry codes: (i) 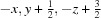; (ii) 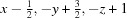; (iii) 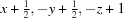; (iv) 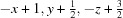.

**Table 2:** Backbone torsion angles ω, Φ, Ψ (°) for the two tripeptide fragments

Pro1	ω1	C20—C19—N3—C15	170.3 (3)
	φ1	C19—N3—C15—C14	−66.2 (4)
	ψ1	N3—C15—C14—N2	152.4 (3)
Phe2	ω2	C15—C14—N2—C6	165.7 (3)
	φ2	C14—N2—C6—C5	−68.9 (4)
	ψ2	N2—C6—C5—N1	−48.1 (4)
Ala3	ω3	C6—C5—N1—C3	−174.4 (3)
	φ3	C5—N1—C3—C2	−145.1 (3)
	ψ3	N1—C3—C2—O1	158.6 (3)
Pro4	ω4	C31—C32—N4—C36	−169.3 (3)
	φ4	C32—N4—C36—C37	−58.8 (3)
	ψ4	N4—C36—C37—N5	−32.5 (4)
Phe5	ω5	C36—C37—N5—C38	−173.0 (3)
	φ5	C37—N5—C38—C46	−96.4 (3)
	ψ5	N5—C38—C46—N6	22.6 (4)
Ala6	ω6	C38—C46—N6—C47	169.3 (3)
	φ6	C46—N6—C47—C49	−52.1 (4)
	ψ6	N6—C47—C49—O10	−31.8 (4)

**Table 3:** Experimental details

Crystal data	
Chemical formula	C_50_H_56_N_6_O_10_·0.5H_2_O
*M*_r_	910.01
Crystal system, space group	Orthorhombic, *P*2_1_2_1_2_1_
Temperature (K)	100
*a*, *b*, *c* (Å)	9.9955 (5), 15.8364 (7), 31.1356 (14)
*V* (Å^3^)	4928.5 (4)
*Z*	4
Radiation type	Mo *K*α
μ (mm^−1^)	0.09
Crystal size (mm)	0.28 × 0.2 × 0.15

Data collection	
Diffractometer	Bruker D8 Quest CMOS
Absorption correction	Multi-scan (*SADABS*; Bruker, 2013 ▸)
*T*_min_, *T*_max_	0.695, 0.745
No. of measured, independent and observed [*I* > 2σ(*I*)] reflections	43863, 10447, 8909
*R*_int_	0.038
(sin θ/λ)_max_ (Å^−1^)	0.634

Refinement	
*R*[*F* ^2^ > 2σ(*F* ^2^)], *wR*(*F* ^2^), *S*	0.048, 0.130, 1.06
No. of reflections	10447
No. of parameters	621
No. of restraints	39
H-atom treatment	H-atom parameters constrained
Δρ_max_, Δρ_min_ (e Å^−3^)	1.05, −0.17

Computer programs: *APEX2* and *SAINT* (Bruker, 2013 ▸), *olex2.solve* (Bourhis *et al.*, 2015 ▸), *SHELXL* (Sheldrick, 2015 ▸) and *OLEX2* (Dolomanov *et al.*, 2009 ▸).

## supplementary crystallographic information

### Crystal data

**Table:**

C_50_H_56_N_6_O_10_·0.5H_2_O	*D*_x_ = 1.226 Mg m^−^^3^
*M**_r_* = 910.01	Mo *K*α radiation, λ = 0.71073 Å
Orthorhombic, *P*2_1_2_1_2_1_	Cell parameters from 9965 reflections
*a* = 9.9955 (5) Å	θ = 3.1–26.5°
*b* = 15.8364 (7) Å	µ = 0.09 mm^−^^1^
*c* = 31.1356 (14) Å	*T* = 100 K
*V* = 4928.5 (4) Å^3^	Prism, clear light yellow
*Z* = 4	0.28 × 0.2 × 0.15 mm
*F*(000) = 1932	

### Data collection

**Table:**

Bruker D8 Quest CMOS diffractometer	8909 reflections with *I* > 2σ(*I*)
φ and ω scans	*R*_int_ = 0.038
Absorption correction: multi-scan (SADABS; Bruker, 2013)	θ_max_ = 26.8°, θ_min_ = 2.9°
*T*_min_ = 0.695, *T*_max_ = 0.745	*h* = −12→12
43863 measured reflections	*k* = −20→20
10447 independent reflections	*l* = −39→32

### Refinement

**Table:**

Refinement on *F*^2^	Hydrogen site location: mixed
Least-squares matrix: full	H-atom parameters constrained
*R*[*F*^2^ > 2σ(*F*^2^)] = 0.048	*w* = 1/[σ^2^(*F*_o_^2^) + (0.0688*P*)^2^ + 1.586*P*] where *P* = (*F*_o_^2^ + 2*F*_c_^2^)/3
*wR*(*F*^2^) = 0.130	(Δ/σ)_max_ < 0.001
*S* = 1.06	Δρ_max_ = 1.05 e Å^−^^3^
10447 reflections	Δρ_min_ = −0.17 e Å^−^^3^
621 parameters	Absolute structure: Flack *x* determined using 3422 quotients [(*I*^+^)-(*I*^-^)]/[(*I*^+^)+(*I*^-^)] (Parsons et al., 2013)
39 restraints	Absolute structure parameter: 0.1 (3)
Primary atom site location: iterative	

### Special details

**Table:**

Geometry. All esds (except the esd in the dihedral angle between two l.s. planes) are estimated using the full covariance matrix. The cell esds are taken into account individually in the estimation of esds in distances, angles and torsion angles; correlations between esds in cell parameters are only used when they are defined by crystal symmetry. An approximate (isotropic) treatment of cell esds is used for estimating esds involving l.s. planes.

### Fractional atomic coordinates and isotropic or equivalent isotropic displacement parameters (Å^2^)

**Table:**

	*x*	*y*	*z*	*U*_iso_*/*U*_eq_	Occ. (<1)
O8	0.5783 (2)	0.35379 (15)	0.61299 (7)	0.0290 (5)	
O5	0.1677 (2)	0.52485 (14)	0.62202 (7)	0.0300 (5)	
O6	0.1527 (2)	0.33804 (15)	0.71036 (7)	0.0297 (5)	
O7	0.1823 (2)	0.20517 (14)	0.61792 (8)	0.0328 (5)	
O9	0.7545 (2)	0.24915 (17)	0.69600 (8)	0.0368 (6)	
O4	0.3271 (2)	0.67992 (16)	0.56633 (8)	0.0358 (6)	
O10	0.5908 (3)	0.18598 (15)	0.65811 (8)	0.0376 (6)	
O3	0.3607 (2)	0.78162 (16)	0.46470 (8)	0.0366 (6)	
O2	0.0132 (3)	0.94017 (16)	0.53066 (9)	0.0458 (7)	
N5	0.2180 (2)	0.34595 (17)	0.61273 (8)	0.0211 (5)	
H5	0.1869	0.3968	0.6182	0.025*	
O1	0.1553 (3)	1.04279 (16)	0.51105 (9)	0.0452 (7)	
N2	0.1356 (3)	0.66860 (16)	0.52816 (8)	0.0251 (6)	
H2	0.0490	0.6587	0.5292	0.030*	
N6	0.4311 (3)	0.32243 (18)	0.66559 (8)	0.0259 (6)	
H6	0.3466	0.3178	0.6731	0.031*	
N3	0.1878 (3)	0.65960 (17)	0.64292 (8)	0.0296 (6)	
N4	−0.0114 (2)	0.37316 (16)	0.66369 (8)	0.0229 (5)	
N1	0.1567 (3)	0.82474 (17)	0.48518 (10)	0.0336 (7)	
H1	0.0797	0.8092	0.4964	0.040*	
C5	0.2483 (3)	0.7659 (2)	0.47812 (10)	0.0255 (7)	
C2	0.1053 (4)	0.9653 (2)	0.50913 (12)	0.0329 (8)	
C49	0.6401 (3)	0.2466 (2)	0.68322 (10)	0.0273 (7)	
C37	0.1444 (3)	0.27814 (19)	0.62330 (10)	0.0234 (6)	
C19	0.2126 (3)	0.5774 (2)	0.64748 (10)	0.0236 (6)	
C46	0.4634 (3)	0.34112 (19)	0.62472 (10)	0.0226 (6)	
C40	0.2546 (3)	0.3822 (2)	0.52165 (9)	0.0246 (6)	
C38	0.3471 (3)	0.3369 (2)	0.59253 (9)	0.0226 (6)	
H38	0.3491	0.2788	0.5800	0.027*	
C32	0.0613 (3)	0.3867 (2)	0.69939 (10)	0.0247 (7)	
C25	0.2567 (3)	0.52550 (19)	0.72356 (9)	0.0227 (6)	
C20	0.3034 (3)	0.55266 (19)	0.68390 (9)	0.0231 (6)	
C33	−0.1105 (3)	0.4298 (2)	0.64412 (10)	0.0272 (7)	
H33A	−0.0832	0.4896	0.6468	0.033*	
H33B	−0.1999	0.4223	0.6573	0.033*	
C45	0.1501 (3)	0.4389 (2)	0.51655 (10)	0.0284 (7)	
H45	0.1490	0.4896	0.5328	0.034*	
C22	0.5320 (3)	0.5374 (2)	0.70738 (12)	0.0320 (7)	
H22	0.6253	0.5417	0.7020	0.038*	
C31	0.0216 (3)	0.4610 (2)	0.72602 (10)	0.0268 (7)	
C34	−0.1097 (3)	0.4010 (2)	0.59730 (10)	0.0306 (7)	
H34A	−0.1946	0.4158	0.5827	0.037*	
H34B	−0.0341	0.4263	0.5813	0.037*	
C39	0.3631 (3)	0.3978 (2)	0.55481 (9)	0.0254 (7)	
H39A	0.3574	0.4567	0.5653	0.031*	
H39B	0.4521	0.3897	0.5415	0.031*	
C41	0.2561 (3)	0.3086 (2)	0.49701 (10)	0.0292 (7)	
H41	0.3279	0.2698	0.5000	0.035*	
C23	0.4861 (3)	0.5099 (2)	0.74670 (12)	0.0328 (8)	
H23	0.5481	0.4948	0.7685	0.039*	
C43	0.0489 (4)	0.3485 (2)	0.46349 (11)	0.0332 (8)	
H43	−0.0213	0.3372	0.4437	0.040*	
C8	0.1648 (4)	0.6529 (2)	0.40682 (10)	0.0312 (7)	
C14	0.2050 (3)	0.6776 (2)	0.56506 (10)	0.0265 (7)	
C44	0.0472 (4)	0.4222 (2)	0.48784 (11)	0.0316 (8)	
H44	−0.0245	0.4612	0.4848	0.038*	
C26	0.1115 (3)	0.5259 (2)	0.73614 (9)	0.0255 (7)	
C7	0.1065 (3)	0.6452 (2)	0.45158 (10)	0.0306 (7)	
H7A	0.0829	0.5854	0.4570	0.037*	
H7B	0.0232	0.6788	0.4531	0.037*	
C47	0.5353 (3)	0.3100 (2)	0.69728 (10)	0.0269 (7)	
H47	0.5805	0.3654	0.7025	0.032*	
C6	0.2021 (3)	0.6751 (2)	0.48676 (10)	0.0265 (7)	
H6A	0.2823	0.6374	0.4869	0.032*	
C42	0.1532 (4)	0.2917 (2)	0.46815 (10)	0.0326 (8)	
H42	0.1546	0.2412	0.4517	0.039*	
C21	0.4407 (3)	0.5586 (2)	0.67586 (11)	0.0286 (7)	
H21	0.4714	0.5773	0.6486	0.034*	
C30	−0.1064 (4)	0.4624 (3)	0.74307 (11)	0.0395 (9)	
H30	−0.1668	0.4179	0.7366	0.047*	
C36	0.0037 (3)	0.2945 (2)	0.63950 (10)	0.0252 (7)	
H36	−0.0263	0.2459	0.6576	0.030*	
C24	0.3505 (3)	0.5042 (2)	0.75457 (10)	0.0293 (7)	
H24	0.3207	0.4852	0.7819	0.035*	
C9	0.1242 (4)	0.7179 (2)	0.37993 (11)	0.0383 (8)	
H9	0.0580	0.7568	0.3893	0.046*	
C35	−0.0937 (3)	0.3060 (2)	0.60144 (11)	0.0307 (7)	
H35A	−0.0560	0.2815	0.5748	0.037*	
H35B	−0.1808	0.2787	0.6075	0.037*	
C4	0.1237 (4)	0.9350 (2)	0.43102 (12)	0.0353 (8)	
H4A	0.1771	0.9056	0.4092	0.053*	
H4B	0.1296	0.9961	0.4264	0.053*	
H4C	0.0302	0.9170	0.4288	0.053*	
C29	−0.1478 (4)	0.5278 (3)	0.76945 (13)	0.0531 (12)	
H29	−0.2350	0.5271	0.7816	0.064*	
C15	0.1186 (4)	0.6907 (2)	0.60477 (10)	0.0317 (8)	
H15	0.0294	0.6629	0.6014	0.038*	
C27	0.0672 (3)	0.5929 (3)	0.76131 (12)	0.0390 (9)	
H27	0.1256	0.6387	0.7672	0.047*	
C13	0.2600 (4)	0.5956 (2)	0.39230 (11)	0.0355 (8)	
H13	0.2886	0.5510	0.4105	0.043*	
C3	0.1767 (3)	0.9136 (2)	0.47531 (12)	0.0334 (8)	
H3	0.2745	0.9266	0.4764	0.040*	
C12	0.3135 (4)	0.6032 (3)	0.35134 (12)	0.0440 (9)	
H12	0.3776	0.5634	0.3414	0.053*	
C10	0.1802 (5)	0.7262 (3)	0.33924 (12)	0.0451 (10)	
H10	0.1539	0.7717	0.3212	0.054*	
C28	−0.0625 (4)	0.5933 (3)	0.77791 (14)	0.0561 (13)	
H28	−0.0917	0.6393	0.7951	0.067*	
C11	0.2732 (4)	0.6690 (3)	0.32496 (12)	0.0465 (10)	
H11	0.3100	0.6745	0.2970	0.056*	
C18	0.2329 (4)	0.7290 (2)	0.67105 (12)	0.0388 (9)	
H18C	0.3232	0.7490	0.6628	0.047*	0.746 (11)
H18D	0.2340	0.7114	0.7016	0.047*	0.746 (11)
H18A	0.3292	0.7245	0.6782	0.047*	0.254 (11)
H18B	0.1798	0.7323	0.6978	0.047*	0.254 (11)
C48	0.4734 (4)	0.2800 (3)	0.73931 (11)	0.0402 (9)	
H48A	0.4047	0.3202	0.7485	0.060*	
H48B	0.5430	0.2761	0.7614	0.060*	
H48C	0.4326	0.2243	0.7351	0.060*	
C50	0.6830 (5)	0.1212 (2)	0.64429 (13)	0.0473 (10)	
H50A	0.7417	0.1438	0.6219	0.071*	
H50B	0.6327	0.0730	0.6328	0.071*	
H50C	0.7372	0.1026	0.6688	0.071*	
C16	0.1028 (5)	0.7859 (3)	0.61383 (13)	0.0511 (11)	
H16C	0.0117	0.8053	0.6062	0.061*	0.746 (11)
H16D	0.1688	0.8190	0.5971	0.061*	0.746 (11)
H16A	0.1091	0.8189	0.5869	0.061*	0.254 (11)
H16B	0.0156	0.7978	0.6276	0.061*	0.254 (11)
C1	0.0909 (6)	1.0984 (3)	0.54255 (14)	0.0606 (13)	
H1A	−0.0051	1.1017	0.5365	0.091*	
H1B	0.1303	1.1549	0.5407	0.091*	
H1C	0.1046	1.0756	0.5715	0.091*	
O11	0.3543 (5)	0.0810 (3)	0.6507 (2)	0.0484 (14)	0.5
H11A	0.3164	0.1111	0.6307	0.073*	0.5
H11B	0.4078	0.1161	0.6636	0.073*	0.5
C17B	0.1282 (8)	0.7962 (4)	0.6631 (2)	0.0526 (18)	0.746 (11)
H17A	0.1625	0.8533	0.6700	0.063*	0.746 (11)
H17B	0.0459	0.7854	0.6799	0.063*	0.746 (11)
C17A	0.204 (2)	0.8050 (9)	0.6403 (5)	0.047 (3)	0.254 (11)
H17C	0.1801	0.8555	0.6574	0.057*	0.254 (11)
H17D	0.2847	0.8182	0.6233	0.057*	0.254 (11)

### Atomic displacement parameters (Å^2^)

**Table:**

	*U*^11^	*U*^22^	*U*^33^	*U*^12^	*U*^13^	*U*^23^
O8	0.0180 (11)	0.0412 (13)	0.0278 (12)	0.0043 (9)	0.0010 (9)	−0.0014 (10)
O5	0.0355 (13)	0.0288 (11)	0.0256 (11)	−0.0027 (10)	−0.0080 (10)	0.0001 (10)
O6	0.0248 (11)	0.0390 (12)	0.0254 (11)	0.0010 (10)	−0.0052 (9)	0.0020 (10)
O7	0.0321 (12)	0.0245 (12)	0.0419 (14)	0.0017 (10)	0.0016 (11)	−0.0055 (10)
O9	0.0242 (12)	0.0512 (15)	0.0351 (13)	0.0127 (11)	−0.0037 (10)	0.0015 (11)
O4	0.0274 (13)	0.0468 (14)	0.0333 (12)	0.0080 (11)	−0.0101 (10)	−0.0035 (11)
O10	0.0392 (14)	0.0353 (13)	0.0384 (14)	0.0094 (11)	−0.0051 (11)	−0.0034 (11)
O3	0.0253 (12)	0.0438 (14)	0.0407 (13)	−0.0030 (11)	0.0031 (10)	−0.0034 (12)
O2	0.0446 (16)	0.0348 (14)	0.0578 (17)	−0.0044 (12)	0.0116 (14)	−0.0067 (13)
N5	0.0173 (12)	0.0239 (13)	0.0222 (12)	0.0018 (10)	−0.0022 (10)	−0.0012 (10)
O1	0.0634 (18)	0.0328 (13)	0.0393 (14)	−0.0167 (13)	−0.0107 (13)	−0.0015 (12)
N2	0.0224 (13)	0.0277 (13)	0.0252 (13)	0.0021 (11)	−0.0046 (11)	0.0002 (11)
N6	0.0172 (12)	0.0391 (15)	0.0213 (12)	0.0049 (11)	−0.0014 (10)	0.0013 (12)
N3	0.0371 (15)	0.0299 (14)	0.0218 (13)	0.0084 (12)	−0.0079 (12)	−0.0024 (12)
N4	0.0197 (13)	0.0271 (14)	0.0220 (12)	−0.0007 (10)	−0.0024 (10)	−0.0025 (11)
N1	0.0206 (13)	0.0251 (14)	0.0550 (18)	−0.0025 (11)	0.0028 (13)	0.0033 (14)
C5	0.0217 (15)	0.0329 (17)	0.0219 (14)	−0.0012 (13)	−0.0060 (12)	−0.0029 (13)
C2	0.0330 (18)	0.0254 (17)	0.0403 (19)	−0.0030 (15)	−0.0114 (16)	0.0037 (15)
C49	0.0243 (16)	0.0335 (17)	0.0242 (15)	0.0084 (13)	−0.0011 (13)	0.0054 (14)
C37	0.0239 (15)	0.0243 (16)	0.0219 (14)	−0.0009 (12)	−0.0043 (12)	−0.0028 (13)
C19	0.0226 (15)	0.0288 (16)	0.0194 (14)	0.0006 (12)	0.0019 (12)	−0.0003 (13)
C46	0.0190 (15)	0.0261 (16)	0.0228 (15)	0.0057 (12)	0.0002 (12)	−0.0012 (13)
C40	0.0254 (15)	0.0301 (16)	0.0182 (14)	−0.0049 (13)	0.0025 (12)	0.0018 (13)
C38	0.0183 (14)	0.0271 (16)	0.0224 (14)	0.0047 (12)	−0.0007 (12)	−0.0025 (13)
C32	0.0182 (14)	0.0348 (17)	0.0211 (15)	−0.0048 (13)	0.0005 (12)	0.0031 (14)
C25	0.0175 (14)	0.0295 (16)	0.0211 (14)	0.0007 (12)	−0.0011 (12)	−0.0041 (13)
C20	0.0238 (15)	0.0234 (15)	0.0220 (15)	0.0045 (12)	−0.0012 (12)	−0.0034 (13)
C33	0.0184 (14)	0.0346 (17)	0.0284 (16)	0.0043 (13)	−0.0021 (13)	−0.0027 (14)
C45	0.0318 (17)	0.0260 (16)	0.0273 (16)	−0.0054 (13)	−0.0040 (14)	0.0028 (14)
C22	0.0175 (15)	0.0378 (19)	0.0405 (19)	0.0006 (14)	0.0039 (14)	0.0035 (16)
C31	0.0184 (15)	0.0426 (19)	0.0193 (14)	−0.0038 (14)	−0.0020 (12)	−0.0056 (14)
C34	0.0210 (15)	0.046 (2)	0.0248 (16)	0.0036 (15)	−0.0076 (13)	0.0003 (15)
C39	0.0227 (15)	0.0329 (17)	0.0207 (14)	−0.0008 (13)	−0.0006 (12)	0.0007 (13)
C41	0.0299 (17)	0.0336 (18)	0.0240 (15)	−0.0007 (14)	0.0034 (14)	−0.0016 (14)
C23	0.0194 (16)	0.045 (2)	0.0335 (18)	0.0016 (14)	−0.0076 (14)	0.0080 (16)
C43	0.0337 (18)	0.043 (2)	0.0233 (16)	−0.0124 (16)	−0.0046 (14)	0.0028 (15)
C8	0.0371 (19)	0.0316 (17)	0.0249 (15)	−0.0057 (15)	−0.0093 (14)	−0.0030 (14)
C14	0.0281 (16)	0.0242 (16)	0.0271 (16)	0.0065 (13)	−0.0074 (13)	0.0013 (13)
C44	0.0310 (17)	0.0341 (18)	0.0298 (17)	−0.0034 (14)	−0.0072 (14)	0.0076 (15)
C26	0.0168 (14)	0.0420 (18)	0.0176 (14)	−0.0009 (13)	−0.0022 (12)	−0.0043 (14)
C7	0.0336 (18)	0.0280 (16)	0.0302 (17)	−0.0032 (14)	−0.0070 (14)	−0.0014 (14)
C47	0.0197 (15)	0.0386 (19)	0.0225 (15)	0.0069 (13)	−0.0028 (12)	0.0011 (14)
C6	0.0278 (16)	0.0278 (16)	0.0240 (15)	0.0054 (13)	−0.0035 (13)	−0.0026 (13)
C42	0.0368 (18)	0.0384 (18)	0.0227 (15)	−0.0092 (15)	0.0032 (14)	−0.0059 (14)
C21	0.0258 (16)	0.0329 (17)	0.0271 (16)	−0.0027 (13)	0.0069 (13)	0.0037 (14)
C30	0.0230 (17)	0.065 (2)	0.0305 (17)	−0.0115 (17)	−0.0001 (14)	−0.0164 (18)
C36	0.0216 (15)	0.0269 (16)	0.0270 (16)	−0.0033 (12)	−0.0037 (13)	−0.0027 (13)
C24	0.0242 (16)	0.0427 (19)	0.0210 (15)	−0.0012 (14)	−0.0001 (13)	0.0050 (14)
C9	0.053 (2)	0.0340 (18)	0.0280 (17)	−0.0026 (17)	−0.0114 (16)	−0.0049 (16)
C35	0.0195 (15)	0.043 (2)	0.0298 (17)	−0.0022 (14)	−0.0056 (13)	−0.0112 (15)
C4	0.0323 (18)	0.0298 (17)	0.044 (2)	−0.0040 (14)	0.0060 (16)	0.0004 (16)
C29	0.0184 (17)	0.096 (3)	0.045 (2)	−0.004 (2)	0.0057 (16)	−0.036 (2)
C15	0.0359 (19)	0.0358 (18)	0.0234 (15)	0.0135 (15)	−0.0040 (14)	0.0006 (14)
C27	0.0215 (16)	0.059 (2)	0.0369 (19)	−0.0037 (16)	−0.0039 (14)	−0.0240 (19)
C13	0.042 (2)	0.0325 (18)	0.0319 (17)	−0.0042 (16)	−0.0101 (16)	−0.0050 (15)
C3	0.0238 (16)	0.0261 (16)	0.050 (2)	−0.0059 (13)	−0.0012 (15)	0.0029 (16)
C12	0.039 (2)	0.056 (2)	0.037 (2)	−0.0027 (18)	−0.0057 (16)	−0.0170 (19)
C10	0.064 (3)	0.044 (2)	0.0269 (17)	−0.008 (2)	−0.0122 (18)	0.0006 (17)
C28	0.0237 (18)	0.091 (3)	0.054 (2)	0.000 (2)	0.0028 (17)	−0.046 (3)
C11	0.053 (2)	0.063 (3)	0.0238 (17)	−0.017 (2)	−0.0078 (17)	−0.0028 (18)
C18	0.050 (2)	0.0321 (18)	0.0340 (18)	0.0109 (16)	−0.0127 (17)	−0.0093 (15)
C48	0.0300 (18)	0.066 (3)	0.0249 (17)	0.0155 (18)	−0.0011 (14)	0.0071 (18)
C50	0.068 (3)	0.0342 (19)	0.039 (2)	0.0199 (19)	−0.003 (2)	0.0003 (17)
C16	0.074 (3)	0.043 (2)	0.036 (2)	0.033 (2)	−0.003 (2)	−0.0010 (18)
C1	0.113 (4)	0.032 (2)	0.037 (2)	−0.014 (2)	−0.006 (2)	−0.0101 (18)
O11	0.023 (2)	0.048 (3)	0.074 (4)	0.004 (2)	−0.008 (3)	−0.001 (3)
C17B	0.077 (4)	0.044 (3)	0.037 (3)	0.028 (3)	−0.012 (3)	−0.015 (2)
C17A	0.074 (7)	0.029 (6)	0.040 (6)	0.015 (6)	−0.010 (6)	−0.019 (5)

### Geometric parameters (Å, º)

**Table:**

O8—C46	1.221 (4)	C8—C9	1.388 (5)
O5—C19	1.234 (4)	C8—C13	1.390 (5)
O6—C32	1.243 (4)	C14—C15	1.523 (5)
O7—C37	1.227 (4)	C44—H44	0.9500
O9—C49	1.211 (4)	C26—C27	1.391 (5)
O4—C14	1.221 (4)	C7—H7A	0.9900
O10—C49	1.333 (4)	C7—H7B	0.9900
O10—C50	1.445 (4)	C7—C6	1.529 (4)
O3—C5	1.224 (4)	C47—H47	1.0000
O2—C2	1.207 (4)	C47—C48	1.523 (5)
N5—H5	0.8800	C6—H6A	1.0000
N5—C37	1.343 (4)	C42—H42	0.9500
N5—C38	1.443 (4)	C21—H21	0.9500
O1—C2	1.326 (4)	C30—H30	0.9500
O1—C1	1.466 (5)	C30—C29	1.384 (6)
N2—H2	0.8800	C36—H36	1.0000
N2—C14	1.350 (4)	C36—C35	1.544 (4)
N2—C6	1.454 (4)	C24—H24	0.9500
N6—H6	0.8800	C9—H9	0.9500
N6—C46	1.346 (4)	C9—C10	1.391 (5)
N6—C47	1.448 (4)	C35—H35A	0.9900
N3—C19	1.332 (4)	C35—H35B	0.9900
N3—C15	1.460 (4)	C4—H4A	0.9800
N3—C18	1.476 (4)	C4—H4B	0.9800
N4—C32	1.345 (4)	C4—H4C	0.9800
N4—C33	1.469 (4)	C4—C3	1.515 (5)
N4—C36	1.463 (4)	C29—H29	0.9500
N1—H1	0.8800	C29—C28	1.369 (6)
N1—C5	1.325 (4)	C15—H15	1.0000
N1—C3	1.454 (4)	C15—C16	1.542 (5)
C5—C6	1.534 (5)	C27—H27	0.9500
C2—C3	1.513 (5)	C27—C28	1.395 (5)
C49—C47	1.515 (4)	C13—H13	0.9500
C37—C36	1.516 (4)	C13—C12	1.388 (5)
C19—C20	1.505 (4)	C3—H3	1.0000
C46—C38	1.536 (4)	C12—H12	0.9500
C40—C45	1.386 (5)	C12—C11	1.387 (6)
C40—C39	1.518 (4)	C10—H10	0.9500
C40—C41	1.396 (5)	C10—C11	1.372 (6)
C38—H38	1.0000	C28—H28	0.9500
C38—C39	1.528 (4)	C11—H11	0.9500
C32—C31	1.492 (5)	C18—H18C	0.9900
C25—C20	1.388 (4)	C18—H18D	0.9900
C25—C26	1.503 (4)	C18—H18A	0.9900
C25—C24	1.387 (4)	C18—H18B	0.9900
C20—C21	1.398 (5)	C18—C17B	1.513 (6)
C33—H33A	0.9900	C18—C17A	1.566 (15)
C33—H33B	0.9900	C48—H48A	0.9800
C33—C34	1.527 (4)	C48—H48B	0.9800
C45—H45	0.9500	C48—H48C	0.9800
C45—C44	1.388 (5)	C50—H50A	0.9800
C22—H22	0.9500	C50—H50B	0.9800
C22—C23	1.378 (5)	C50—H50C	0.9800
C22—C21	1.382 (5)	C16—H16C	0.9900
C31—C26	1.402 (5)	C16—H16D	0.9900
C31—C30	1.385 (5)	C16—H16A	0.9900
C34—H34A	0.9900	C16—H16B	0.9900
C34—H34B	0.9900	C16—C17B	1.563 (7)
C34—C35	1.519 (5)	C16—C17A	1.335 (17)
C39—H39A	0.9900	C1—H1A	0.9800
C39—H39B	0.9900	C1—H1B	0.9800
C41—H41	0.9500	C1—H1C	0.9800
C41—C42	1.391 (5)	O11—H11A	0.8701
C23—H23	0.9500	O11—H11B	0.8694
C23—C24	1.380 (5)	C17B—H17A	0.9900
C43—H43	0.9500	C17B—H17B	0.9900
C43—C44	1.392 (5)	C17A—H17C	0.9900
C43—C42	1.386 (5)	C17A—H17D	0.9900
C8—C7	1.515 (5)		
			
C49—O10—C50	116.8 (3)	C41—C42—H42	120.0
C37—N5—H5	119.4	C43—C42—C41	119.9 (3)
C37—N5—C38	121.2 (3)	C43—C42—H42	120.0
C38—N5—H5	119.4	C20—C21—H21	119.8
C2—O1—C1	114.9 (3)	C22—C21—C20	120.3 (3)
C14—N2—H2	119.6	C22—C21—H21	119.8
C14—N2—C6	120.8 (3)	C31—C30—H30	119.5
C6—N2—H2	119.6	C29—C30—C31	121.1 (4)
C46—N6—H6	119.9	C29—C30—H30	119.5
C46—N6—C47	120.1 (3)	N4—C36—C37	114.4 (2)
C47—N6—H6	119.9	N4—C36—H36	109.5
C19—N3—C15	120.3 (3)	N4—C36—C35	103.3 (2)
C19—N3—C18	127.4 (3)	C37—C36—H36	109.5
C15—N3—C18	112.1 (3)	C37—C36—C35	110.5 (3)
C32—N4—C33	127.6 (3)	C35—C36—H36	109.5
C32—N4—C36	120.4 (3)	C25—C24—H24	119.2
C36—N4—C33	112.1 (2)	C23—C24—C25	121.6 (3)
C5—N1—H1	118.3	C23—C24—H24	119.2
C5—N1—C3	123.4 (3)	C8—C9—H9	120.0
C3—N1—H1	118.3	C8—C9—C10	120.1 (4)
O3—C5—N1	123.2 (3)	C10—C9—H9	120.0
O3—C5—C6	121.8 (3)	C34—C35—C36	104.4 (3)
N1—C5—C6	115.0 (3)	C34—C35—H35A	110.9
O2—C2—O1	124.6 (4)	C34—C35—H35B	110.9
O2—C2—C3	124.6 (3)	C36—C35—H35A	110.9
O1—C2—C3	110.8 (3)	C36—C35—H35B	110.9
O9—C49—O10	124.4 (3)	H35A—C35—H35B	108.9
O9—C49—C47	122.4 (3)	H4A—C4—H4B	109.5
O10—C49—C47	113.0 (3)	H4A—C4—H4C	109.5
O7—C37—N5	123.4 (3)	H4B—C4—H4C	109.5
O7—C37—C36	119.5 (3)	C3—C4—H4A	109.5
N5—C37—C36	116.9 (3)	C3—C4—H4B	109.5
O5—C19—N3	121.5 (3)	C3—C4—H4C	109.5
O5—C19—C20	121.9 (3)	C30—C29—H29	120.2
N3—C19—C20	116.6 (3)	C28—C29—C30	119.6 (4)
O8—C46—N6	123.0 (3)	C28—C29—H29	120.2
O8—C46—C38	121.6 (3)	N3—C15—C14	110.2 (3)
N6—C46—C38	115.2 (3)	N3—C15—H15	111.1
C45—C40—C39	120.8 (3)	N3—C15—C16	103.3 (3)
C45—C40—C41	119.1 (3)	C14—C15—H15	111.1
C41—C40—C39	120.1 (3)	C14—C15—C16	109.9 (3)
N5—C38—C46	112.8 (2)	C16—C15—H15	111.1
N5—C38—H38	106.2	C26—C27—H27	119.7
N5—C38—C39	111.5 (2)	C26—C27—C28	120.6 (4)
C46—C38—H38	106.2	C28—C27—H27	119.7
C39—C38—C46	113.3 (3)	C8—C13—H13	119.8
C39—C38—H38	106.2	C12—C13—C8	120.4 (4)
O6—C32—N4	121.7 (3)	C12—C13—H13	119.8
O6—C32—C31	122.1 (3)	N1—C3—C2	108.2 (3)
N4—C32—C31	116.2 (3)	N1—C3—C4	111.1 (3)
C20—C25—C26	123.7 (3)	N1—C3—H3	109.0
C24—C25—C20	117.9 (3)	C2—C3—C4	110.4 (3)
C24—C25—C26	118.2 (3)	C2—C3—H3	109.0
C25—C20—C19	123.3 (3)	C4—C3—H3	109.0
C25—C20—C21	120.7 (3)	C13—C12—H12	120.1
C21—C20—C19	116.1 (3)	C11—C12—C13	119.8 (4)
N4—C33—H33A	111.3	C11—C12—H12	120.1
N4—C33—H33B	111.3	C9—C10—H10	119.8
N4—C33—C34	102.2 (2)	C11—C10—C9	120.4 (4)
H33A—C33—H33B	109.2	C11—C10—H10	119.8
C34—C33—H33A	111.3	C29—C28—C27	120.2 (4)
C34—C33—H33B	111.3	C29—C28—H28	119.9
C40—C45—H45	119.7	C27—C28—H28	119.9
C40—C45—C44	120.6 (3)	C12—C11—H11	120.0
C44—C45—H45	119.7	C10—C11—C12	120.1 (4)
C23—C22—H22	120.4	C10—C11—H11	120.0
C23—C22—C21	119.2 (3)	N3—C18—H18C	111.3
C21—C22—H22	120.4	N3—C18—H18D	111.3
C26—C31—C32	122.2 (3)	N3—C18—H18A	112.0
C30—C31—C32	118.1 (3)	N3—C18—H18B	112.0
C30—C31—C26	119.6 (3)	N3—C18—C17B	102.4 (3)
C33—C34—H34A	111.3	N3—C18—C17A	98.7 (6)
C33—C34—H34B	111.3	H18C—C18—H18D	109.2
H34A—C34—H34B	109.2	H18A—C18—H18B	109.7
C35—C34—C33	102.4 (3)	C17B—C18—H18C	111.3
C35—C34—H34A	111.3	C17B—C18—H18D	111.3
C35—C34—H34B	111.3	C17A—C18—H18A	112.0
C40—C39—C38	110.2 (3)	C17A—C18—H18B	112.0
C40—C39—H39A	109.6	C47—C48—H48A	109.5
C40—C39—H39B	109.6	C47—C48—H48B	109.5
C38—C39—H39A	109.6	C47—C48—H48C	109.5
C38—C39—H39B	109.6	H48A—C48—H48B	109.5
H39A—C39—H39B	108.1	H48A—C48—H48C	109.5
C40—C41—H41	119.7	H48B—C48—H48C	109.5
C42—C41—C40	120.5 (3)	O10—C50—H50A	109.5
C42—C41—H41	119.7	O10—C50—H50B	109.5
C22—C23—H23	119.8	O10—C50—H50C	109.5
C22—C23—C24	120.4 (3)	H50A—C50—H50B	109.5
C24—C23—H23	119.8	H50A—C50—H50C	109.5
C44—C43—H43	120.1	H50B—C50—H50C	109.5
C42—C43—H43	120.1	C15—C16—H16C	110.7
C42—C43—C44	119.8 (3)	C15—C16—H16D	110.7
C9—C8—C7	120.1 (3)	C15—C16—H16A	110.8
C9—C8—C13	119.2 (3)	C15—C16—H16B	110.8
C13—C8—C7	120.6 (3)	C15—C16—C17B	105.4 (3)
O4—C14—N2	123.0 (3)	H16C—C16—H16D	108.8
O4—C14—C15	122.5 (3)	H16A—C16—H16B	108.8
N2—C14—C15	114.4 (3)	C17B—C16—H16C	110.7
C45—C44—C43	120.1 (3)	C17B—C16—H16D	110.7
C45—C44—H44	120.0	C17A—C16—C15	104.9 (6)
C43—C44—H44	120.0	C17A—C16—H16A	110.8
C31—C26—C25	123.9 (3)	C17A—C16—H16B	110.8
C27—C26—C25	117.3 (3)	O1—C1—H1A	109.5
C27—C26—C31	118.8 (3)	O1—C1—H1B	109.5
C8—C7—H7A	108.9	O1—C1—H1C	109.5
C8—C7—H7B	108.9	H1A—C1—H1B	109.5
C8—C7—C6	113.2 (3)	H1A—C1—H1C	109.5
H7A—C7—H7B	107.8	H1B—C1—H1C	109.5
C6—C7—H7A	108.9	H11A—O11—H11B	104.4
C6—C7—H7B	108.9	C18—C17B—C16	101.5 (4)
N6—C47—C49	113.0 (3)	C18—C17B—H17A	111.5
N6—C47—H47	108.4	C18—C17B—H17B	111.5
N6—C47—C48	109.6 (3)	C16—C17B—H17A	111.5
C49—C47—H47	108.4	C16—C17B—H17B	111.5
C49—C47—C48	108.9 (3)	H17A—C17B—H17B	109.3
C48—C47—H47	108.4	C18—C17A—H17C	109.6
N2—C6—C5	111.1 (3)	C18—C17A—H17D	109.6
N2—C6—C7	109.1 (3)	C16—C17A—C18	110.2 (11)
N2—C6—H6A	108.6	C16—C17A—H17C	109.6
C5—C6—H6A	108.6	C16—C17A—H17D	109.6
C7—C6—C5	110.7 (3)	H17C—C17A—H17D	108.1
C7—C6—H6A	108.6		

### Hydrogen-bond geometry (Å, º)

**Table:**

*D*—H···*A*	*D*—H	H···*A*	*D*···*A*	*D*—H···*A*
O11—H11*B*···O10	0.87	2.15	2.899 (6)	145
O11—H11*A*···O7	0.87	2.03	2.804 (6)	145
C29—H29···O11^i^	0.95	2.57	3.339 (7)	138
N5—H5···O5	0.88	2.04	2.892 (3)	163
N2—H2···O3^ii^	0.88	2.11	2.867 (4)	143
C6—H6*A*···O2^iii^	1.00	2.67	3.647 (4)	165
C4—H4*C*···O4^ii^	0.98	2.55	3.480 (4)	159
C27—H27···O9^iv^	0.95	2.41	3.327 (5)	162
C1—H1*B*···O7^v^	0.98	2.59	3.033 (5)	108
C1—H1*C*···O7^v^	0.98	2.63	3.033 (5)	105

Symmetry codes: (i) −*x*, *y*+1/2, −*z*+3/2; (ii) *x*−1/2, −*y*+3/2, −*z*+1; (iii) *x*+1/2, −*y*+1/2, −*z*+1; (iv) −*x*+1, *y*+1/2, −*z*+3/2; (v) *x*, *y*+1, *z*.
